# The Amelioration of Grazing through Physiological Integration by a Clonal Dune Plant

**DOI:** 10.3390/plants12040724

**Published:** 2023-02-06

**Authors:** Jonathan P. Evans, Shelby Meckstroth, Julie Garai

**Affiliations:** 1 Department of Biology, University of the South, Sewanee, TN 37383, USA; 2 Department of Mathematics and Computer Science, University of the South, Sewanee, TN 37383, USA

**Keywords:** *Hydrocotyle bonariensis*, coastal dunes, feral horses, clonal integration, rhizome, defoliation

## Abstract

Rhizomatous growth and associated physiological integration can allow a clonal dune species to potentially compensate for the selective removal of leaves associated with herbivory. *Hydrocotyle bonariensis* is a rhizomatous clonal plant species that is abundant in the coastal dune environments of the southeastern United States that are inhabited by large feral horse populations. *H. bonariensis* has been shown to integrate resources among ramets within extensive clones as an adaptation to resource heterogeneity in sandy soils. In this study, we hypothesized that clonal integration is a mechanism that promotes *H. bonariensis* persistence in these communities, despite high levels of herbivory by feral horses. In a field experiment, we used exclosures to test for herbivory in *H. bonariensis* over a four-month period. We found that feral horses utilized *H. bonariensis* as a food species, and that while grazing will suppress clonal biomass, *H. bonariensis* is able to maintain populations in a high grazing regime with and without competition present. We then conducted an experiment in which portions of *H. bonariensis* clones were clipped to simulate different levels of grazing. Half of the clones were severed to eliminate the possibility of integration. We found that after 12 weeks, the mean number of leaves and ramets increased as the grazing level increased, for integrated clones. Integrated clones had significantly increased biomass production compared to the severed equivalents. Our research suggests that rhizomatous growth and physiological integration are traits that allow clonal plant species to maintain populations and to tolerate grazing in coastal dune environments.

## 1. Introduction

Clonal plants are an important component of many ecosystems, including coastal dunes, and their success in these environments is associated with their ability to occupy space and to persist over time through the proliferation and maintenance of potentially physiologically independent shoots or ramets [1,2,3,4]. Some clonal species generate ramets through the extension of underground stems called rhizomes, and ramets that remain interconnected via rhizomes have the potential to share resources throughout the clone; in doing so, this allows a clone to become physiologically integrated [5,6,7]. In heterogeneous environments, clonal integration can promote the growth and survival of an entire clone by supporting ramets experiencing resource limitation [4,8,9]. Through active source–sink relationships, ramets in unfavorable patches may proliferate at rates that are comparable to ramets in favorable patches [7,10].

Clonal plants are common in heavily grazed ecosystems. Grazing can negatively affect a plant’s fitness and productivity by altering plant growth and biomass allocation [11]. Being grazed thereby decreases the plant’s photosynthetic area and reproductive structures [12,13,14], and ultimately impacts on survival [15]. Clones spanning a patchy grazing regime can potentially compensate for lost tissue within a clone through the transport of photosynthate from ungrazed to grazed portions of a clone, allowing for the production of new photosynthetic tissue [16]. By spreading the risk of genet death among ramets within a clone, clonal plants can increase the probability of survival in grazed environments [17,18].

Feral ungulate grazing is a widespread phenomenon on the coastal barrier islands of the Eastern United States [19,20], and it can dramatically alter the structure, composition, and productivity of coastal dune plant communities [21,22,23,24,25]. *Hydrocotyle bonariensis* (dune pennywort), a clonal dune herb, is abundant in coastal dune ecosystems in the southeastern United States that have large feral horse populations [23]. Feral horse activity (grazing, trampling, and excretion) promotes early successional dune communities that are dominated by rhizomatous clonal plant species, such as *H. bonariensis* [23,26]. *H. bonariensis* clones are able to utilize the translocation and integration of resources to promote sexual reproduction and clonal expansion [1,7,27], possibly giving them an advantage in dune environments with horses. Specifically, *H. bonariensis* has been shown to integrate resources across extensive branched rhizome systems spanning areas of patchy water, light, nitrogen, and salt distribution within dune soils [7,27,28]. In *H. bonariensis*, nitrogen transport is facilitated by the existence of strong water potential gradients that are set up within the clone through evapotranspiration. Nitrogen is moved passively in the transpirational flow to ramets in water-deficient areas, before being converted to organic compounds in the leaves. The passive bulk transport of the N in the transpiration stream may effectively couple the integration of these two resources within the clones growing in open dune environments [7].

In this study, we experimentally tested whether clonal integration in *H. bonariensis* allowed for reproductive success, clonal expansion, and biomass accumulation in response to variable grazing regimes. Specifically, we tested the following hypotheses: (1) *H. bonariensis* is able to maintain populations in a high grazing regime with and without competition present (Field Grazing Assessment); (2) *H. bonariensis* can integrate as a compensatory mechanism in response to the defoliation that occurs with grazing (Simulated Grazing and Integration Experiment).

## 2. Methods

### 2.1. Species Description

*Hydrocotyle bonariensis* (Apiaceae) is a rhizomatous plant that is common in coastal dune ecosystems of the southeastern United States [29]. The species forms extensive clonal populations in the dune ridge and in the swale communities on these islands [30]. Clones may be composed of thousands of interconnected ramets spanning over 100 square meters of dune area [7]. The rhizomes grow linearly and have solitary leaves composed of a petiole and a peltate blade, and roots at regularly spaced nodes, which represent the location of ramets (the smallest potential physiologically independent units) within the clone [27]. At each ramet, along with the leaf and roots, there is also a branch meristem, and opposite the leaf, there is an inflorescence bud. Each branch meristem can produce new rhizome branches, which gives the clone a path to facilitate the translocation of resources between ramets.

### 2.2. Field Grazing Assessment

This experiment was conducted in a *Hydrocotyle bonariensis* population located in an upland coastal dune habitat at the far western side of Rachel Carson National Estuarine Research Reserve near Beaufort, North Carolina. This environment was actively grazed by a resident feral horse population [31]. Five replicates of paired 20 m^2^ plots were randomly located within this population. *H. bonariensis* represented approximately 60–70% cover in each of these plots, with additional species representing < 20% cover. One plot in each pair was surrounded by 2.5 m tall cattle fencing, creating an exclosure to prevent herbivory by large mammals, while the adjacent paired plot was subject to grazing. One half of the area in each plot was randomly assigned to the removal of all competition from other plant species (other than *H. bonariensis*). This was performed at the onset of the experiment, and was repeated seven times at regular intervals. The elimination of competition consisted of removing both the above and belowground portions of the plants. This design structure results in five blocks with four subplots, each being assigned a treatment combination: grazing with competition (unmanipulated condition), grazing without competition, no grazing with competition, and no grazing without competition.

Within each subplot, a 2 m^2^ sampling area was established, consisting of a 4 × 4 grid of 0.25 m^2^ quadrats. All living plant material (above and below ground) was excavated from a 0.05 m^2^ area at the center of a set of four randomly chosen quadrats, and separated into two groups (*H. bonariensis* and other species). These two groups were then dried and weighed for total biomass determinations. The experiment was run for 175 days (June through October). Plant material was excavated following this process at both the beginning and end of the experiment, allowing for the change in plant biomass (beginning–end) to be measured.

### 2.3. Simulated Grazing and Integration Experiment

All *H. bonariensis* material used in the experiment was derived from a single clone originally sampled from Shackleford Banks, NC. The clonal stock was propagated in a common environment (outdoor, sand-filled trays), several months prior to the start of the experiment, to eliminate pre-treatment variation among the replicates. Dune sand used in this study was collected from the Rachel Carson National Estuarine Research Reserve. 

*H. bonariensis* rhizome segments consisting of two ramets and two branches were planted into the center of 3 m long trays filled with fine-grained dune sand. The two rhizome branches were then allowed into the opposite halves of the tray. Trays were made out of 11 cm diameter PVC pipe, one-third of which had been removed lengthwise, with the ends capped to create an open linear container. To test for the effect of grazing, one rhizome branch (“treated branch”) in each tray was subjected to one of three clipping treatments over the course of the experiment: “not grazed”—ramets produced along the branch were not subject to clipping; “low grazed”—50% of randomly chosen leaves and inflorescences were clipped in two-week intervals; “high grazed”—100% of the leaves and inflorescences were clipped in two-week intervals. To test for the effect of integration, half of the clones from each grazing treatment were randomly selected to have the rhizome connection severed between the initial branched ramet pair. This physically separated the two halves of the clone, preventing the potential for resource translocation from one side of the clone to the other. Using this design, Evans (1991) [7] demonstrated the potential of *H. bonariensis* clones to integrate water, nitrogen, and photosynthates across similar branched rhizome networks.

There were 10 replicates for each of the 3 treatments, and trays were arranged in a fully randomized design on outdoor benches at the Duke University Marine Laboratory in Beaufort, NC, subject to full sun. Twice-strength Hoagland’s fertilizer solution [32] was applied once a week, and the trays were watered daily.

After 12 weeks (June–August), the experiment was harvested. At harvest, the rhizome branches from each tray that received the grazing treatment were extracted from the trays, and they were washed and analyzed for the following variables: mean individual leaf area, main rhizome length, total ramet number, total leaf number, and total inflorescence number. The plant material was then dried for 48 h at 60 °C to allow for the determination of total biomass from the dry weight data.

### 2.4. Statistical Analyses

For both experiments, distributional assumptions for the response variables were investigated, and all response variables were best modeled following the lognormal distribution. Residual subject-specific pseudolikelihood was used to estimate the model parameters [33]. Simple effects were further investigated using the least squares means, where interactions were found to be significant. All analyses were performed using PROC GLIMMIX from SAS/STAT software Version 9.4 of the SAS system for Windows [34].

The field assessment involved two analyses: *H. bonariensis* biomass and other plant material biomass. For the *H. bonariensis* biomass, the data were analyzed as a split plot experiment, with the main effects and interactions of weeding and grazing being observed at the whole plot level, and the effects of time (before and after) and interactions of time, with weeding and grazing being observed at the split plot level. For other plant biomass, only plots without weeding were analyzed. The whole plot factor was grazing, and the split plot factor was time (before and after). Because these plots were chosen to be representative of the coastal dune habitat, whole plots and split plots were treated as random effects. 

The simulated grazing and integration experiment was analyzed using an analysis of variance to test the effects of clonal integration and defoliation, including interaction, and the main effects on each of the following response variables for the treated branch: total biomass (TB), number of leaves (L), number of inflorescences (I), number of ramets (R), main rhizome length (MRL), and leaf area (LA). When modeling the leaf area, because several leaves were selected from each clone for measurement, additional model structures were necessary. Because each clone is representative of a larger population of clones, they were measured as a random effect that was replicated at the grazing by the severing level. Leaves were modeled individually, which allowed for an additional investigation into the variability across the levels of grazing and severing with regard to leaf size.

## 3. Results

### 3.1. Field Herbivory Assessment

**Figure 1:**
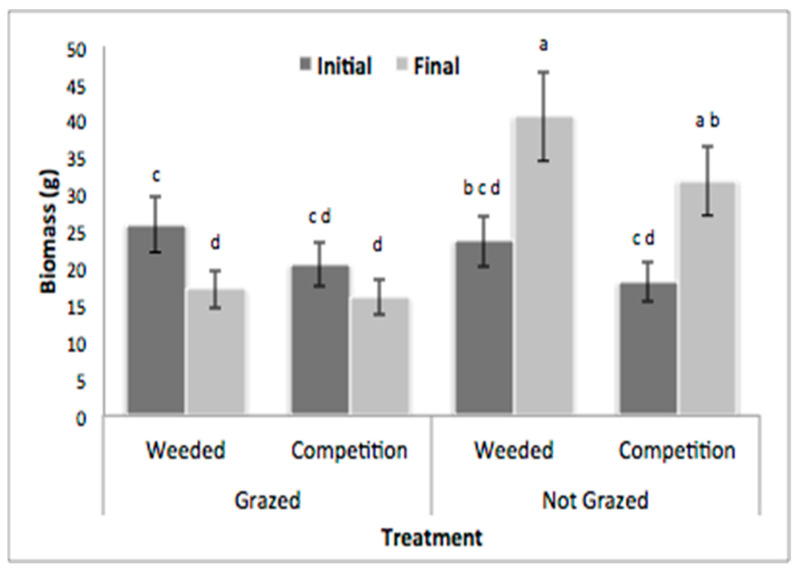
Mean initial and final *H. bonariensis* biomass (grams) for each treatment combination, with (+/−) standard error bars. Where letters above bars differ, this indicates statistically significant differences (Least Squares Means results, alpha = 0.05).

Grazing had a significant effect on *H. bonariensis* biomass over time (*p* = 0.0001; Table 1 (G × T)), with the final average biomass for grazed clones being significantly less than the “not grazed” clones (Least Squares Means; Figure 1). Competition had no significant effect on *H. bonariensis* biomass over time (*p* = 0.5919; Table 1 (C × T)), nor was there a significant competition interaction with grazing and time (*p* = 0.7031; Table 1 (C × G × T)). In the unmanipulated plots (grazed, with competition), there was no significant change in *H. bonariensis* biomass over the course of the experiment (Least Squares Means; Figure 1).

### 3.2. Simulated Grazing and Integration Experiment

#### 3.2.1. Severing Effect

**Figure 2:**
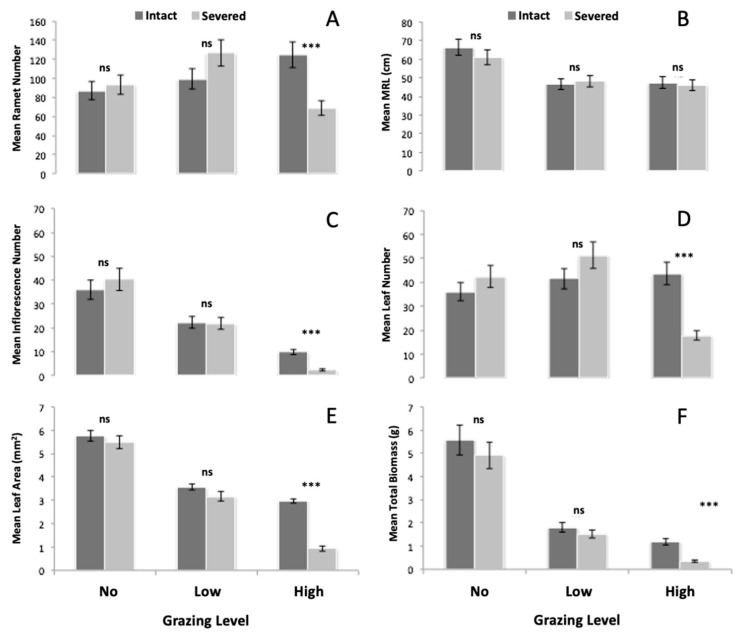
Effects of grazing and integration on mean ramet number (**A**), main rhizome length (MRL) (**B**), inflorescences (**C**), leaf number (**D**), leaf area (**E**), and total biomass (**F**) for each treatment, with least squares means simple effect comparisons and (+/−) standard error bars. Symbols show which means differed between integration treatments for all three grazing levels. Significance levels indicated by ns *p* > 0.05; *** *p* < 0.001.

The ability of *H. bonariensis* clones to respond to grazing through a physiological integration was measured by comparing the intact to the severed treated branches at each grazing level. A significant severing by grazing interaction (S × G) indicates a different response to grazing when the plant was severed. There were no significant differences between the severed and intact clones for any of the six response variables, when both branches received no grazing treatment (*p* > 0.05; Least Square Means; Figure 2A–F). The physical act of severing therefore did not significantly affect the growth of clones spanning uniform conditions.

#### 3.2.2. Biomass Production

Grazing significantly reduced clone biomass production at both low and high grazing levels (*p* < 0.001; Least Square Means; Figure 2). There was a significant severing by grazing interaction for the total biomass of the treated branch (*p* < 0.001; ANOVA Two-way interaction (Total Biomass, S × G); Table 2). However, only in the “high grazed” treatment did intact clones produce more biomass than the severed clones (Mean_Intact_ = 1.17 g and Mean_Severed_ = 0.34 g; *p* < 0.001; Least Squares Means; Figure 2F). 

#### 3.2.3. Leaf and Inflorescence Production

The mean number of leaves produced in the intact grazing treatments was significantly different from the severed grazing treatments (*p* < 0.001; ANOVA two-way interaction ((Leaves, S × G); Table 2). Intact clones produced more leaves in the “high grazed” treatments, and the mean number of leaves produced increased as the grazing level increased for intact clones, (*p* < 0.001; Least Squares Means; Figure 2D). The mean area of individual leaf blades, on the other hand, was significantly less in the high grazed treatment than in the low grazed treatment, in both the intact and severed clones (*p* < 0.001; Least Squares Means; Figure 2E), and in the “high grazed” treatment, intact clones produced more leaves than severed clones (Mean_Intact_ = 44 and Mean_Severed_ = 17 g; *p* < 0.001; Least Squares Means; Figure 2D). In conjunction with the decreased leaf area, as grazing increased, we found that intact, “high grazed” clones produced significantly bigger leaves than severed “high grazed” clones (Mean_Intact_ = 2.97 mm^2^ and Mean_Severed_ = 0.93 mm^2^; *p* < 0.001; Figure 2E, *p* < 0.001; ANOVA two-way interaction (Leaf Area, S × G); Table 2). 

The mean number of inflorescences produced significantly decreased as the grazing level increased, but intact clones produced significantly more inflorescences than the severed clones in the “high grazed” treatment (*p* < 0.001; ANOVA two-way interaction (Inflorescences, S × G); Table 2, Mean_Intact_ = 11 and Mean_Severed_ = 1; *p* < 0.001; Least Squares Means; Figure 2C). 

#### 3.2.4. Ramet Production and Main Rhizome Length

Ramet production in intact grazing treatments was significantly different from the severed treatments (*p* < 0.001; ANOVA two-way interaction (Ramets, S × G); Table 2). Intact clones produced more ramets in the “high grazed” treatments than in the “not grazed” and “low grazed treatments,” and “high grazed”, integrated clones produced more ramets than “high grazed”, severed clones (Mean_Intact_ = 125 and Mean_Severed_ = 68; *p* < 0.001; Least Squares Means; Figure 2A). Grazing caused a reduction in the main rhizome length, but the main rhizome length was not significantly different between the severed and integrated grazing treatments (*p* = 0.665; ANOVA two-way interaction (main rhizome length, S × G); Table 2 and *p* > 0.05; Least Squares Means; Figure 2B). 

## 4. Discussion

In this study, we showed that *H. bonariensis* is utilized as a food species by feral horses in coastal dunes, and that grazing activity resulted in the suppression of growth within clonal populations. However, despite this grazing pressure, populations of *H. bonariensis* maintained a constant clonal biomass over a five-month period, and we found no evidence that the presence of other plant species affected the response of *Hydrocotyle* to grazing over this time period. Our study confirmed our hypothesis that *H. bonariensis* can use integration to ameliorate the effects of grazing by allowing clones to successfully expand into areas experiencing high levels of defoliation, and to maintain ramets under these conditions. Integration allowed for increased biomass production, leaf production, inflorescence production, and the greater proliferation of ramets.

Physiologically integrated clones are hypothesized to be advantageous in environments that have, from the perspective of an individual ramet, high levels of both spatial and temporal resource heterogeneity [4,7]. The presumed adaptive benefit of this kind of integrated response is to allow clones to “average” over spatially and temporally variable resource patches. In turn, this should allow clones to occupy both favorable and unfavorable patches, which should be advantageous since the quality of a given patch may change rapidly in a temporally variable environment [35]. We showed that integration in *H. bonariensis* allowed clones experiencing high grazing to proliferate large networks of interconnected ramets, and therefore, to engage in “risk spreading” [36]. Such a growth strategy should hold an advantage over non-clonal species in unpredictable environments such as sand dunes [1,37].

Sandy soils in early successional dune communities are subject to substrate instability (burial and erosion) and low, patchy nutrient availability, where the primary limiting resource is soil nitrogen [38,39]. Large mammal grazing activity/disturbance can maintain or increase the level of resource heterogeneity that is associated with dune ecosystems [40,41]. Grazing activity can be divided into three types of impacts on plant populations: trampling/soil destabilization, waste deposition, and biomass removal [42]. Trampling can cause direct mortality and the destruction of plant parts (including the severing of roots and rhizomes), and it can cause the destabilization of dune soils by promoting erosion and burial [41]. Through the constant disruption of the soil profile, trampling prevents the accumulation of organic matter within the soil environment [43]. Such changes in soil composition alters nitrogen and moisture availability, which directly affects plant productivity and community composition. Defecation and urination result in scattered, unpredictable point-source increases in nitrogen concentration, which can have immediate toxic effects [44], and can promote the already extremely patchy nitrogen distribution in the soil [45]. Biomass consumption is generally considered to be the most important grazing activity impacting on plant populations [18]. Defoliation under different grazing regimes can cause the premature shedding/loss of plant parts, as well as whole plant mortality [46]. By removing the above-ground plant biomass, grazing directly affects the level of nitrogen stored in plant tissues [46]. Ordinarily, this would increase the rate of nitrogen input to the soil through litter deposition. If grazing inhibits litter accumulation; however, this could represent a net loss or a redistribution of nitrogen within the system. 

Clonal plants (as compared to non-clonal plants) exhibit a variety of mechanisms that allow them to tolerate the effects of grazing, such as an increased level of ramet proliferation, and the ability to draw upon carbohydrate reserves in clonal storage organs, allowing for compensatory growth in response to defoliation [47,48]. In addition to ours, numerous other studies have demonstrated the importance of resource translocation between ramets as a mechanism by which clonal species can respond to defoliation: [49,50,51,52]. Integration has been shown to enhance the clonal response to the other two components of grazing as well: trampling and sand destabilization [53,54], and waste deposition [55].

*Hydrocotyle bonariensis* is similar to other clonal plants in its ability to utilize resource integration as a means of altering its growth form and enhancing survival when subject to conditions of spatial and temporal resource heterogeneity [10,56,57,58]. Previous studies have examined the benefits of resource integration in *H. bonariensis* clones grown across variable resource gradients associated with coastal dune environments [7,27]. The acropetal and basipetal movement of water and nitrogen occurs between rhizome branch systems interconnecting hundreds of ramets within a clone [7]. The extensive movement of water and nitrogen to portions of a clone that are deficient in these resources produces significant net benefits to the clone, in terms of fitness-related traits: total biomass, ramet proliferation, and seed production [1,7,27].

We showed that *H. bonariensis* maintained ramet density under the low grazing regime, and that integrated clones subject to high grazing actually showed higher levels of ramet proliferation compared to the control. In both our field assessment and the severing experiment, we found that there was not a significant compensatory response to defoliation with regard to biomass. We did show, however, that integrated clones had significantly increased biomass production compared to the severed equivalents. In a similar study, Liu et al. (2009) [59] examined the effects of simulated heavy grazing on two clonal, inland dune species from Northern China. They found that integration ameliorated the negative effects of grazing on ramet density, leaf production, and biomass production. In another severing study, Wang et al. (2017) [52] found that clonal integration increased the tolerance of *Iris delavayi* to heavy defoliation by significantly increasing the total biomass, and the rhizome and root biomasses within the affected clone. However, other studies have not found that integration can provide such a benefit [55,60]. The effectiveness of integration is species-specific, and it will likely vary as a function of a number of factors: the scale and spatial pattern of resource heterogeneity within the habitat being grazed, the scale and spatial pattern of grazing activity, the nature of the vascular connections, the directionality of translocation, and the type of resources being transported from the ungrazed to the grazed portions of the clone [7,18,49]. It is also possible that grazing activity can limit the effectiveness of clonal integration through the physical severing of rhizome systems by trampling and consumption [18]. Trampling may also indirectly impact on the effectiveness of integration, by disrupting mycorrhizal facilitation [61].

Within physiologically integrated clones, the morphology and resource capture abilities of individual ramets is determined by both the response of a given ramet to its immediate environment, and its response to resource integration within the clone [1,36,58]. For example, in *H. bonariensis*, ramets grown in low light had significantly larger leaf blade areas than ramets grown in high light, and the blade area was further increased in shaded ramets if they were the recipients of nitrogen and water translocation [1]. In our study, grazing induced *H. bonariensis* ramets to produce substantially smaller leaf blades with shorter petioles, potentially rendering individual leaves to be less apparent to grazers. Integration under the conditions of high grazing; however, somewhat overrode this local response, causing the leaf size to be significantly larger.

While it has been hypothesized that increased rhizome extension may allow clones to escape areas of high herbivory [62], Bittebiere et al. (2020) [18] suggests that this would not represent an effective strategy in response to large mobile grazers, given the relative spatial extent of their grazing activities. We found that main rhizome length actually decreased in response to grazing, and that this variable was not affected by integration. A similar response has been demonstrated in other clonal species as well [18,63,64]. In our field study, we found that the removal of competition did not affect the response of *H. bonariensis* to grazing. Physiological integration has been shown to enhance the competitive abilities of clonal plants in patchy resource environments [65]. *Hydrocotyle bonariensis* has been demonstrated to avoid competition through the selective placement of ramets [66].

Unlike other feral ungulates, feral horse populations are intentionally maintained through active management in a number of National Seashores spanning the coastal barrier islands of the Eastern United States [19]. This presents a unique challenge to US National Park Service managers, since feral horse activity can often compromise other conservation management objectives such as the prevention of dune erosion and the protection of native biodiversity [41,67]. Our study suggests that rhizomatous plant species that are capable of clonal integration may represent a critical group of native species that can tolerate the presence of these introduced animals. 

Wood et al. (1987) [23] showed that in Cape Lookout National Seashore (North Carolina, USA), feral ungulate grazing activity maintained dune plant communities in a perpetual early successional state, where 98% of the aboveground annual plant growth was represented exclusively by two clonal species manifesting extensive rhizomatous growth *(Uniola paniculata* and *H. bonariensis*). However, after 3 years of excluding grazers, rhizomatous clonal species declined to 58% of the aboveground annual plant growth, with other plant life forms becoming abundant. Rhizomatous clonal species may be essential for the maintenance of vegetative cover in coastal dunes that are constantly disturbed by feral animal activity, and their protection should be considered accordingly in the management and restoration of these habitats.

## Figures and Tables

**Table 1:** Statistical effects of grazing and competition on clone biomass. F values and significance levels (* *p* < 0.05; ** *p* < 0.01; *** *p* < 0.001; ns not significant, *p* > 0.05) are given. Degrees of freedom are (1, 16). A significant grazing by competition by time interaction (G × C × T) would indicate a different response to grazing by *H. bonariensis* when plots were weeded.

	Grazing (G)	Competition (C)	Time (T)	G × C	G × T	C × T	G × C × T
Biomass	8.06 **	3.02 *	1.56 ns	0.18 ns	25.11 ***	0.3 ns	0.15 ns

**Table 2:** F values of ANOVA effects of severing and grazing on clone morphological characteristics. Numerator and denominator of degrees of freedom (df) are given. A significant severing by grazing interaction (S × G) indicates a different response to grazing when the plant was severed. Significance levels indicated by, ns *p* > 0.05; * *p* < 0.05; *** *p* < 0.001.

Effect	df	Total Biomass	Leaf Area	Leaves	Inflorescences	Main Rhizome Length	Ramets
S	1.54	28.83 ***	68.58 ***	4.08 *	20.34 ***	0.24 ns	1.15 ns
G	2.54	163.23 ***	164.56 ***	11.82 ***	124.03 ***	14.50 ***	2.44 ns
S × G	2.54	14.68 ***	32.81 ***	17.46 ***	20.62 ***	0.664 ns	34.29 ***

## Data Availability

The data presented in this study are available on request from the corresponding author.
